# Supplementary figures and images for: Evaluation of Monascus purpureus fermentation in dairy sludge-based medium for enhanced production of vibrant red pigment with minimal citrinin content
Source: PLoS One. 2024 Dec 9;19(12):e0315006. doi: 10.1371/journal.pone.0315006 (PMC11627373; doi:10.1371/journal.pone.0315006)

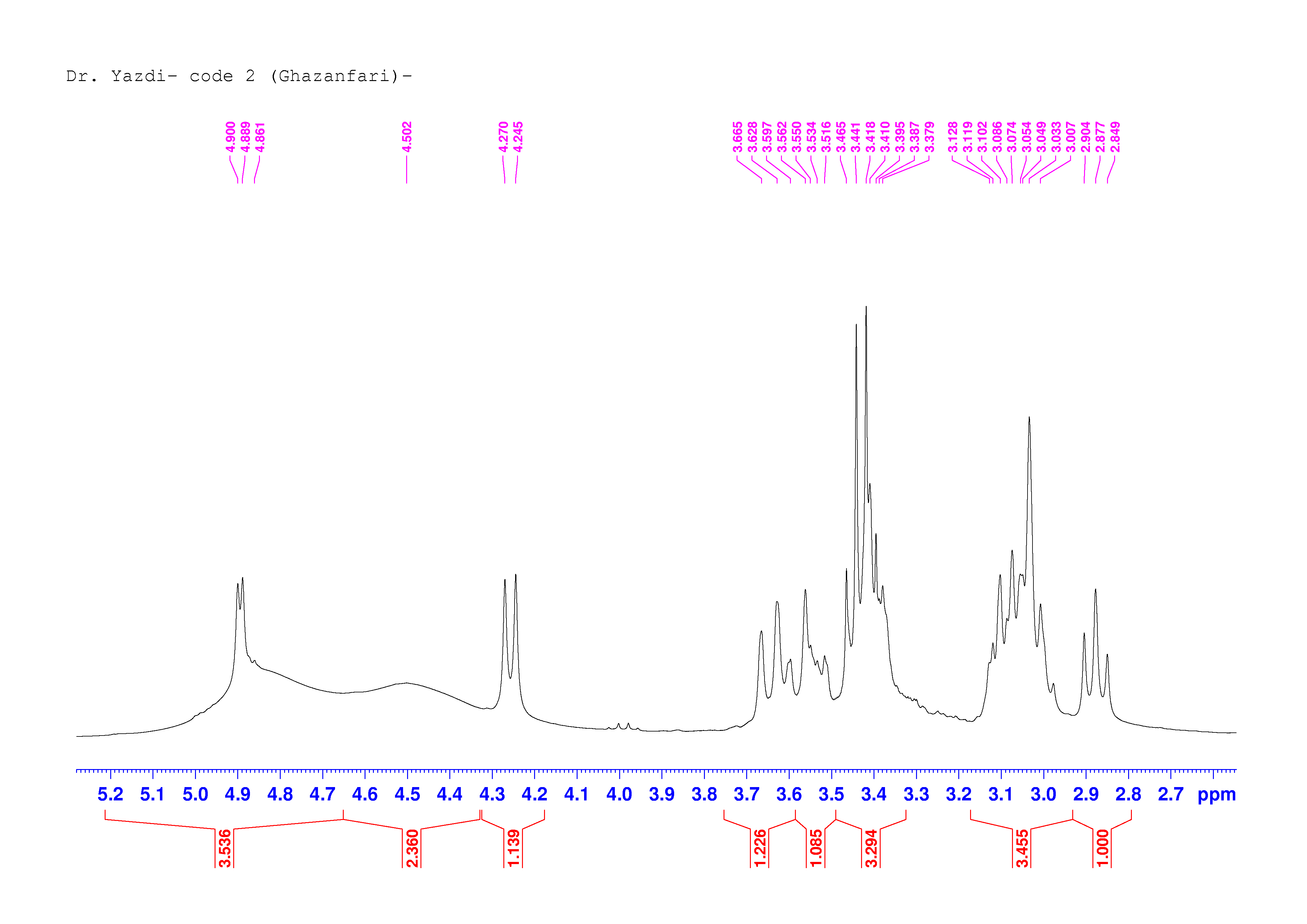

Supplement: S1 File — (ZIP) [file pone.0315006.s001.zip › Dr. Yazdi- code 2 (Ghazanfari)-2.png]

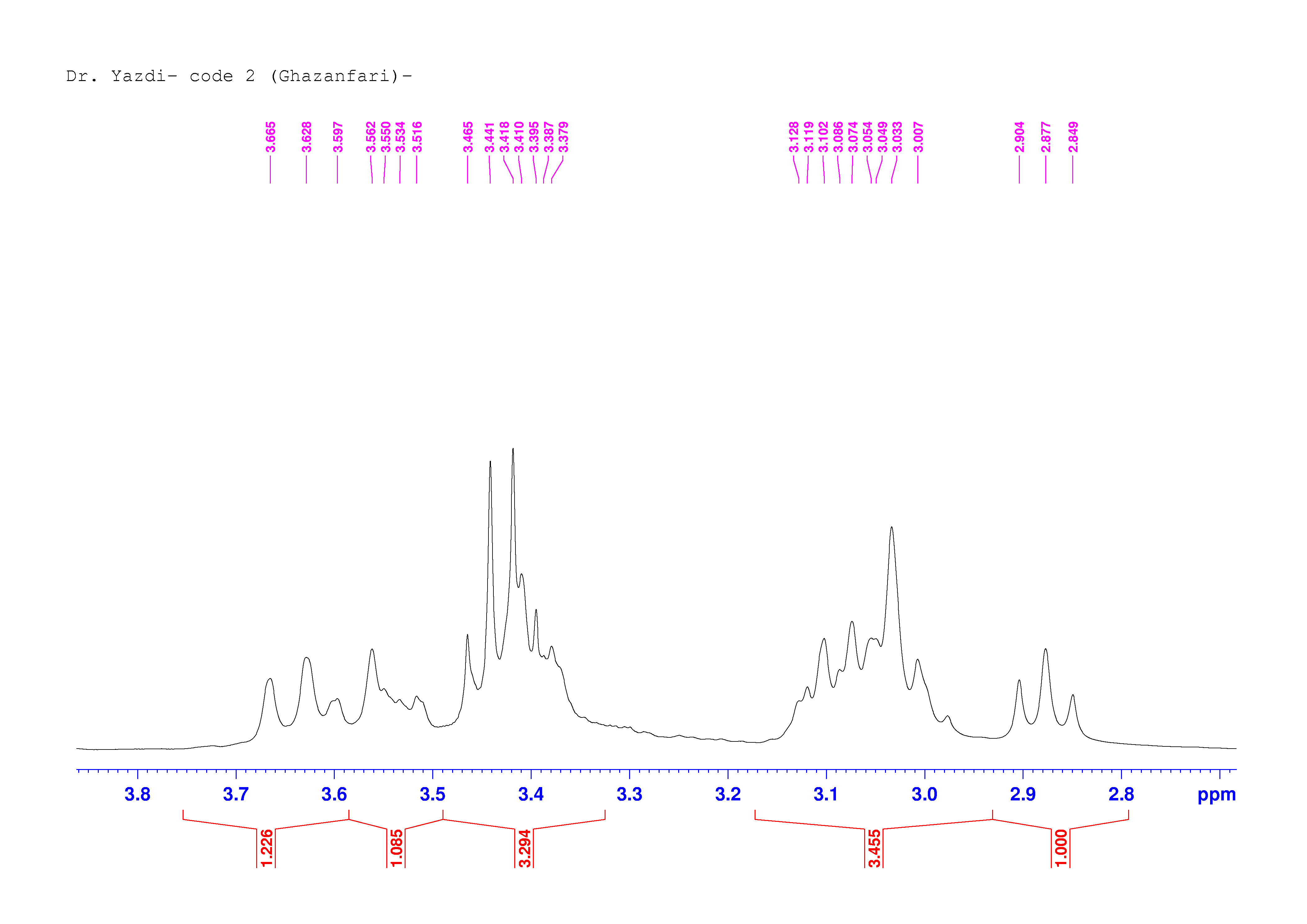

Supplement: S1 File — (ZIP) [file pone.0315006.s001.zip › Dr. Yazdi- code 2 (Ghazanfari)-3.png]

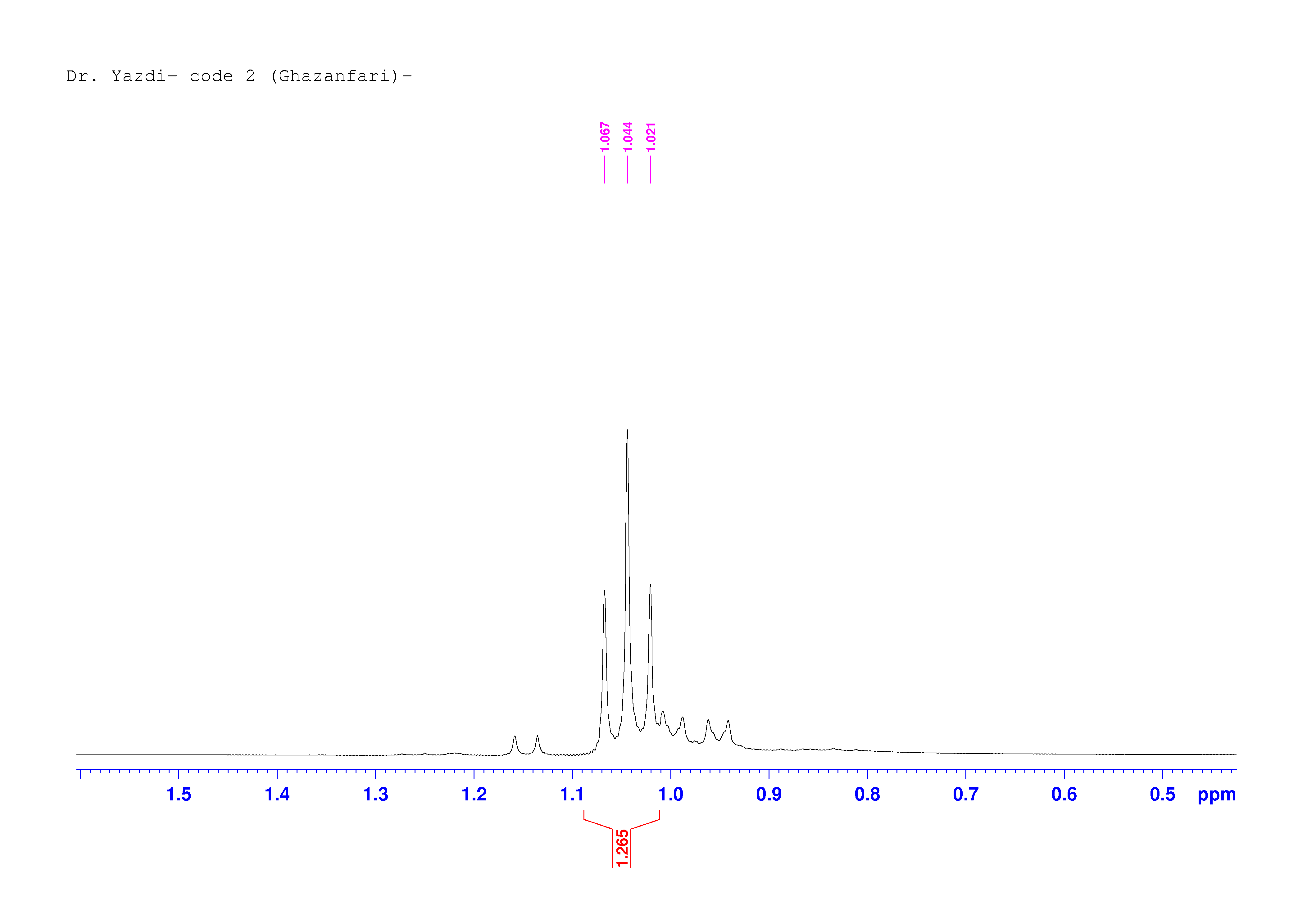

Supplement: S1 File — (ZIP) [file pone.0315006.s001.zip › Dr. Yazdi- code 2 (Ghazanfari)-4.png]

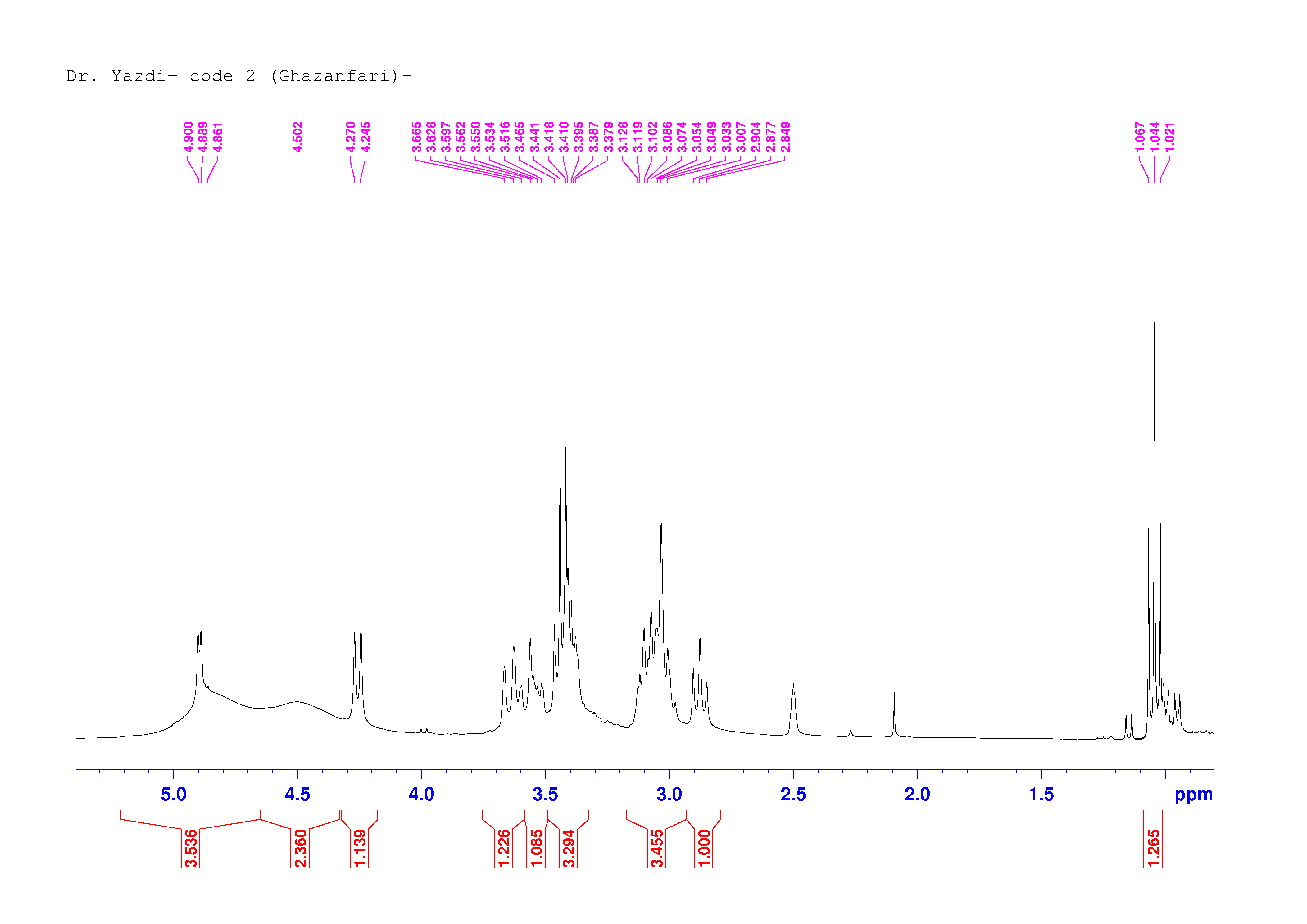

Supplement: S1 File — (ZIP) [file pone.0315006.s001.zip › Dr. Yazdi- code 2 (Ghazanfari)-5.png]

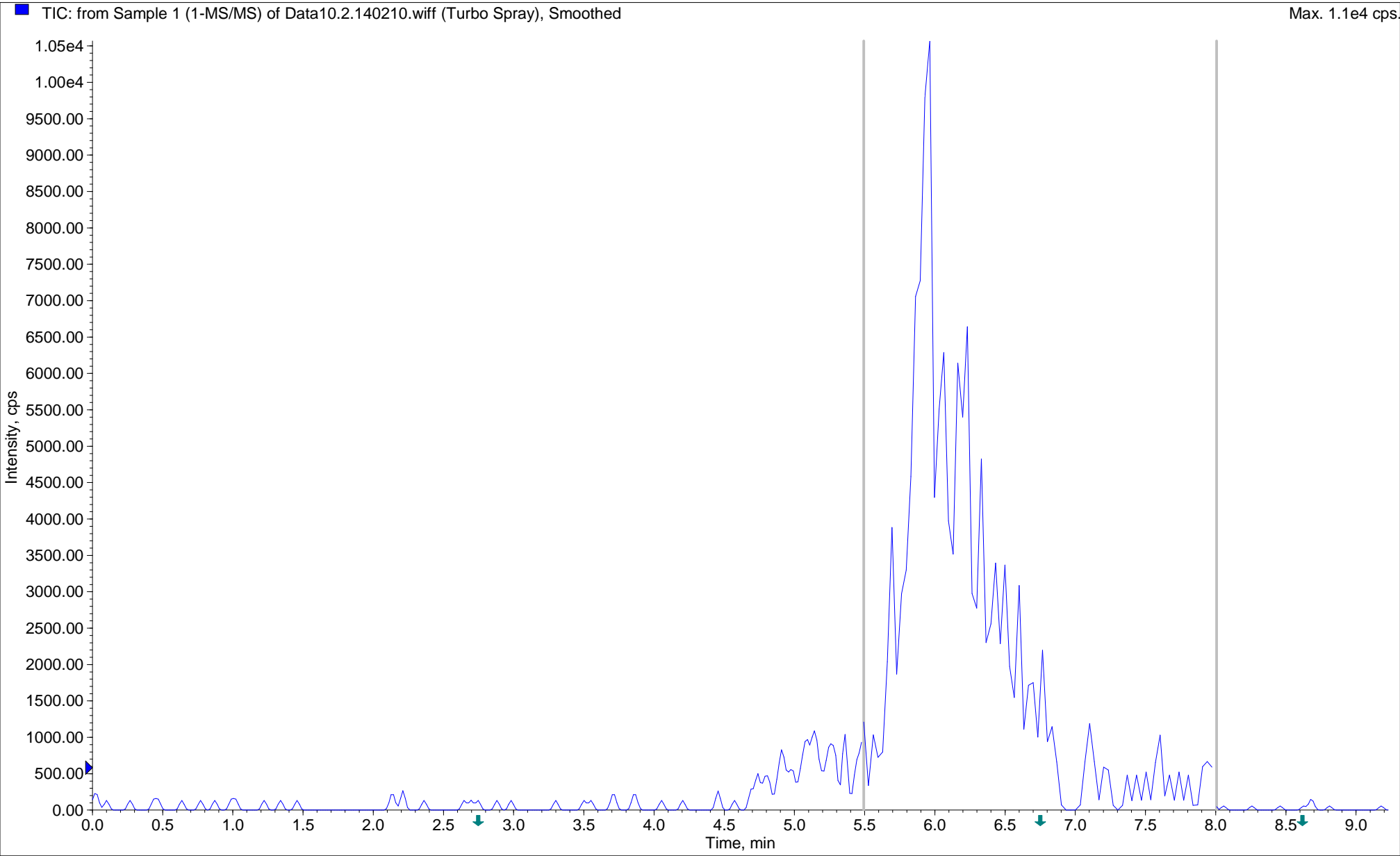

Supplement: S1 File — (ZIP) [file pone.0315006.s001.zip › TIC1.pdf]

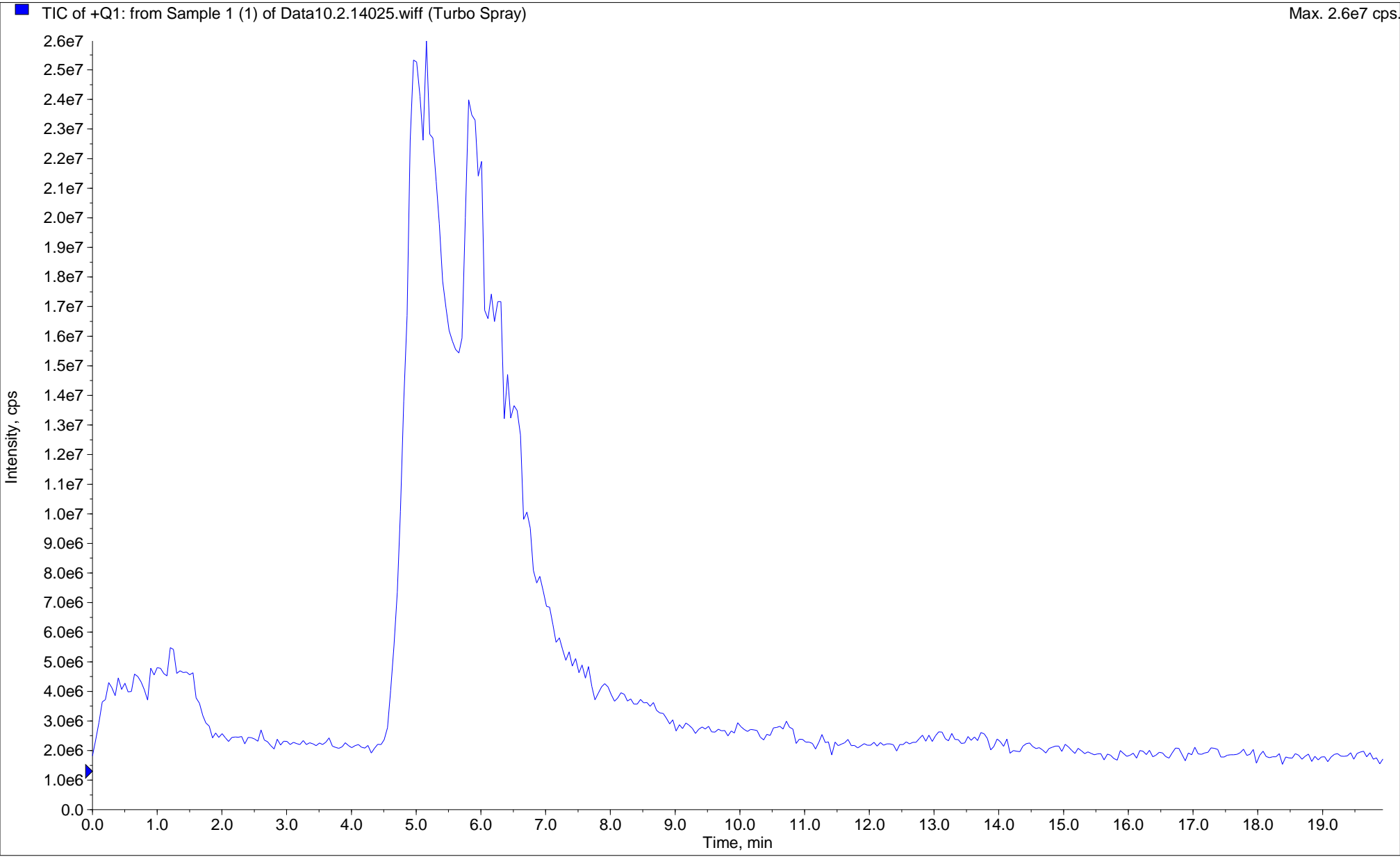

Supplement: S1 File — (ZIP) [file pone.0315006.s001.zip › TIC2.pdf]

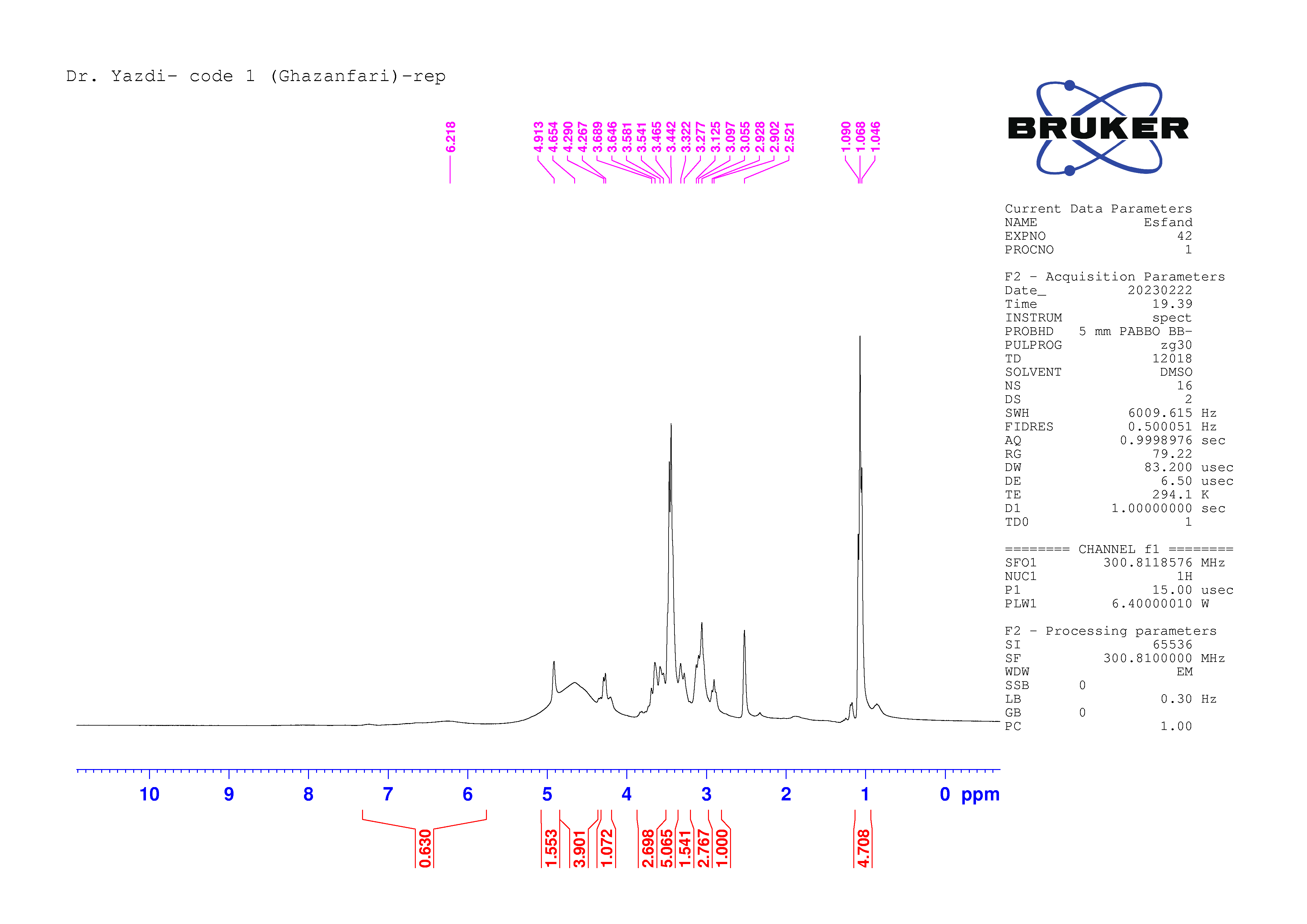

Supplement: S1 File — (ZIP) [file pone.0315006.s001.zip › Dr. Yazdi- code 1 (Ghazanfari)-rep.png]

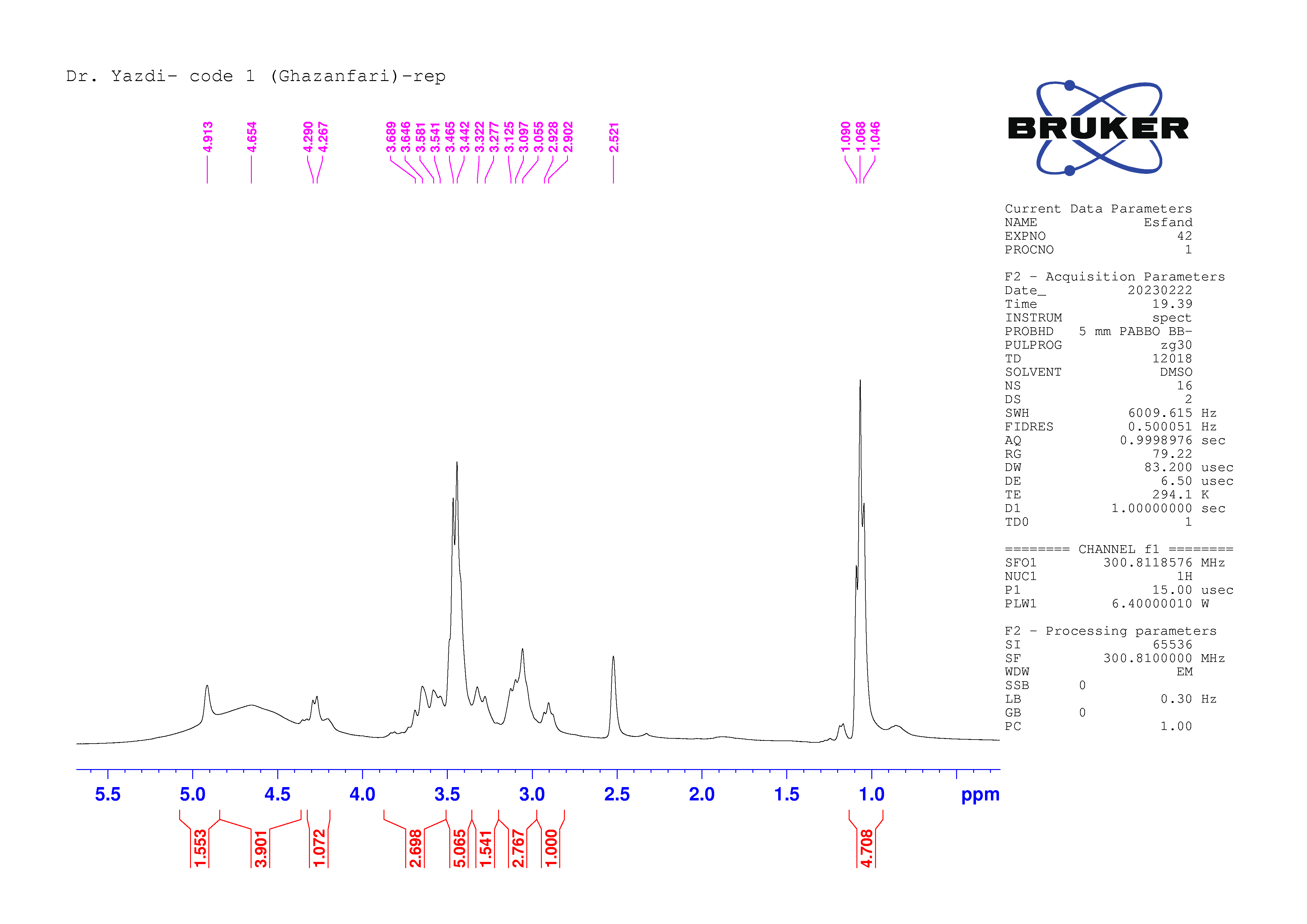

Supplement: S1 File — (ZIP) [file pone.0315006.s001.zip › Dr. Yazdi- code 1 (Ghazanfari)-rep2.png]

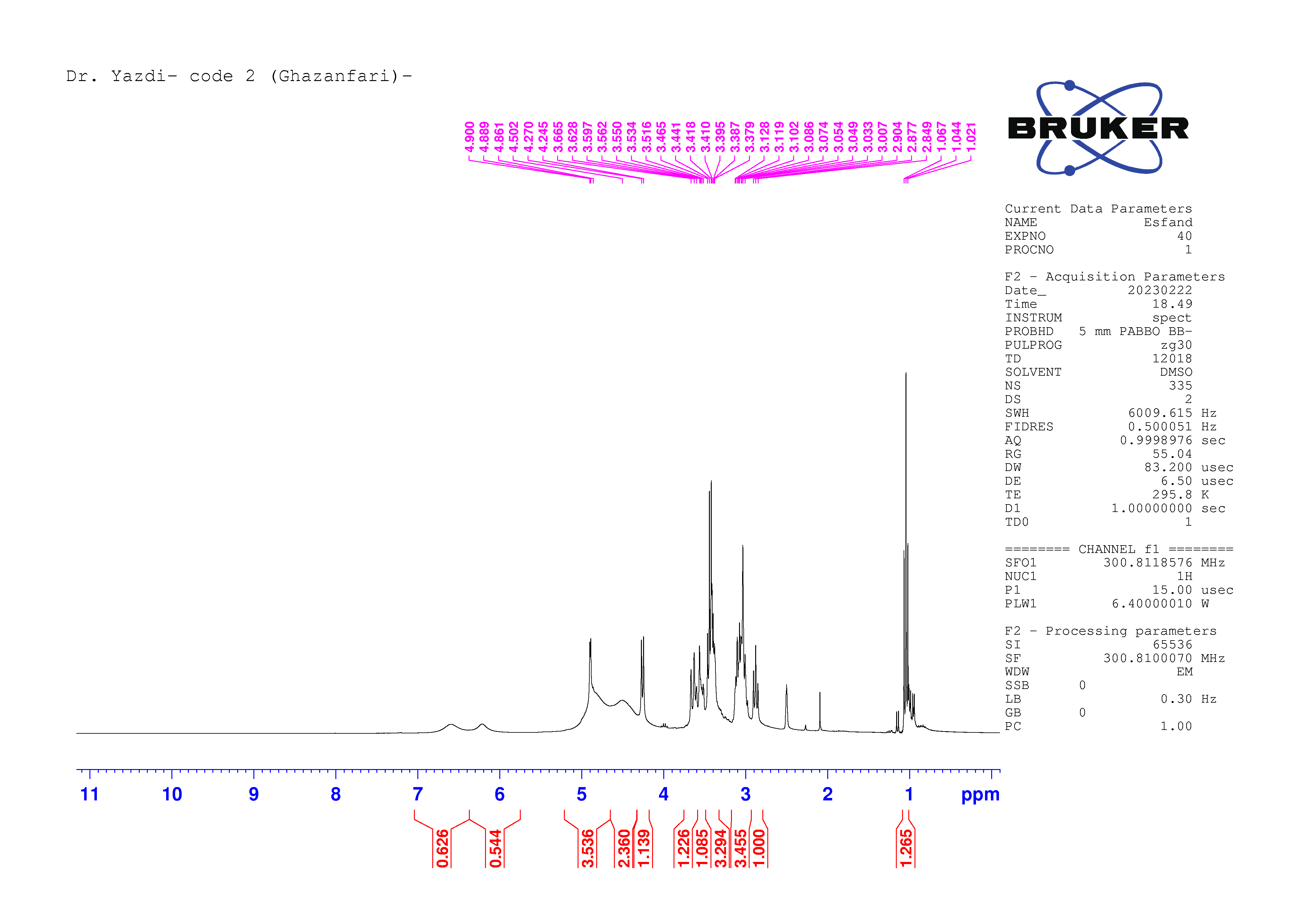

Supplement: S1 File — (ZIP) [file pone.0315006.s001.zip › Dr. Yazdi- code 2 (Ghazanfari)-.png]
